# Clinical significance of the advanced lung cancer inflammation index in gastrointestinal cancer patients: a systematic review and meta-analysis

**DOI:** 10.3389/fonc.2023.1021672

**Published:** 2023-06-19

**Authors:** Hua-Yang Pang, Xiu-Feng Chen, Meng-Hua Yan, Li-Hui Chen, Zhi-Xiong Chen, Shou-Ru Zhang, Hao Sun

**Affiliations:** ^1^ Gastrointestinal Department, Chongqing University Cancer Hospital, Chongqing, China; ^2^ Chongqing Key Laboratory of Translational Research for Cancer Metastasis and Individualized Treatment, Chongqing University Cancer Hospital, Chongqing, China

**Keywords:** gastrointestinal cancer, advanced lung cancer inflammation index, postoperative complications, prognosis, meta-analysis

## Abstract

**Background:**

The advanced lung cancer inflammation index (ALI) has been identified as a scientific and clinical priority in multiple malignancies. The aim of this study is to investigate the value of the ALI before treatment in evaluating postoperative complications (POCs) and survival outcomes in patients with gastrointestinal (GI) cancer.

**Methods:**

Electronic databases including PubMed, Embase and Web of Science were comprehensively reviewed up to June 2022. The endpoints were POCs and survival outcomes. Subgroup analyses and sensitivity analyses were also performed.

**Results:**

Eleven studies including 4417 participants were included. A significant heterogeneity in the ALI cut-off value among studies was observed. Patients in the low ALI group showed increased incidence of POCs (OR=2.02; 95%CI:1.60-2.57; P<0.001; I^2 ^= 0%). In addition, a low ALI was also significantly associated with worse overall survival (HR=1.96; 95%CI: 1.58-2.43; P<0.001; I^2 ^= 64%), which remained consistent in all subgroups based on country, sample size, tumor site, tumor stage, selection method and Newcastle Ottawa Scale score. Moreover, patients in the low ALI group had an obviously decreased disease-free survival compared to these in the high ALI group (HR=1.47; 95%CI: 1.28-1.68; P<0.001; I^2 ^= 0%).

**Conclusion:**

Based on existing evidence, the ALI could act as a valuable predictor of POCs and long-term outcomes in patients with GI cancer. However, the heterogeneity in the ALI cut-off value among studies should be considered when interpreting these findings.

## 1 Introduction

Gastrointestinal (GI) cancers are one of the most common malignancies worldwide, accounting for about 25% of newly diagnosed cancer cases and more than 35% of cancer-related deaths (1). Although significant advances in surgery-based multimodal therapy for gastrointestinal cancers, the clinical efficacy of most of these patients is still poor (2, 3). Consequently, it is essential to develop a useful index to predict the short-term and long-term therapeutic outcomes in GI cancers.

Increasing evidence indicates that cancer-related inflammation and malnutrition status are prevalent in most patients with malignancy, which play an important role in postoperative recovery and prognosis (4, 5). Therefore, inflammation/nutrition-based biomarkers are expected to be valuable predictors of surgical and long-term outcomes. For example, preoperative neutrophil-to-lymphocyte ratio (NLR), based on two blood inflammatory factors, has been reported as a strong indicator for increased postoperative complications (POCs), prolongation of hospital stays and poor survival outcomes in breast cancer (6), lung cancer (7) and GI malignancies (8, 9). Meanwhile, reduced body mass index (BMI) and serum albumin (ALB), which reflect the nutritional status, have also been proven to be associated with adverse therapeutic outcomes in multiple cancers (10–12).

In recent years, a novel biomarker, the advanced lung cancer inflammation index (ALI), which integrates BMI, ALB and NLR (BMI *ALB/NLR), has been reported as a more promising predictor of surgical and long-term outcomes in cancers, because it incorporates multiple nutritional and inflammatory indicators (13, 14). A previous meta-analysis reported that low ALI before surgery indicates poor prognosis in Lung cancer patients (15). Another meta-analysis focusing on the relationship between the ALI and survival outcomes also found a similar conclusion in cancer patients (16). Nevertheless, the role of the ALI in POCs and survival outcomes of GI cancers remains inconclusive and no meta-analysis is available so far. In addition, a number of other studies on the ALI and therapeutic outcomes in GI cancers have been addressed in recent years.

Herein, we performed a systematic review and meta-analysis based on existing evidence to investigate the value of the ALI in POCs and long-term results in GI cancers.

## 2 Methods

The present study was conducted according to the requirements from Preferred Reporting Items for Systematic Reviews and Meta-Analyses (PRISMA) guidelines to identify studies that assess the association of pretreatment ALI with POCs and survival outcomes in GI cancer patients.

### 2.1 Search strategy

Relevant studies from PubMed, Embase and Web of Science were comprehensively examined up to June 30, 2022. Published language was not restricted during the search process. The following combination of key words in title/abstract were used to identify potential studies: [“advanced lung cancer inflammation index” OR “ALI”] AND [“cancer” OR “carcinoma” OR “neoplasm” OR “tumor”] AND [“survival” OR “prognostic” OR “prognosis” OR “mortality” OR “postoperative complications” OR “morbidity”]. In addition, the references of the included studies were scanned for potentially related reports. The search was independently performed by two investigators (HY-P and XF-C).

### 2.2 Inclusion and exclusion criteria

Studies that met the following criteria were included: (1) studies examined the relationship between the pretreatment ALI and POCs or long-term survival of patients with GI cancer; (2) Hazard ratio (HR) with 95% confidence interval (CI) was available or could be calculated based on survival curve; (3) The cutoff value of the ALI was clearly reported.

The exclusion criteria were studies (1) reported as case reports, reviews, letters and conferences; (2) with overlapping data.

### 2.3 Data extraction and quality assessment

Two reviewers (HY-P and XF-C) conducted the data extraction independently and cross-checked all the results. The extracted data included first author, publication year, study interval, country, study design and sample size, selection method, cut-off value, clinicopathological features like age, sex, tumor site and tumor stage, POCs and survival data.

The Newcastle Ottawa Scale (NOS) (17) was used to assess the quality of included studies and a study with NOS score >6 is regarded as of high quality.

### 2.4 Outcomes

In the present study, the primary outcomes were to investigate the relationship between the ALI and POCs or long-term results in patients with GI malignancy. POCs were defined as any morbidity classified as Clavien–Dindo (18) grade I or higher. Long-term outcomes included OS (from the date of surgery to the date of any cause of death) and DFS (from the date of surgery to the date of recurrence or the date of death from any cause).

Of note, since progression-free survival (PFS: from the date of surgery to the date of recurrence or any cause of death), recurrence-free survival (RFS: from the date of surgery to the date of recurrence), cancer-specific survival (CSS: from the date of surgery to the date of cancer-related death) and DFS share the similar endpoints, they were analyzed together as one outcome, DFS (19, 20).

### 2.5 Statistical analysis

The hazard ratios (HRs) and odds ratios (ORs) with their 95% confidence intervals (CIs) were used as the effect size for time-dependent outcomes and dichotomous variables, respectively. For studies that HR with 95%CI was not reported, the indirect data were calculated following the methods reported by Tierney et al. (21) Statistical heterogeneity among studies was assessed using I^2^ statistic. All pooled analyses were performed assuming the random-effects model, which accounts for variance across included studies. Subgroup analysis was used to explore the source of heterogeneity. Sensitivity analysis was conducted to evaluate the credibility of results. Publication bias was evaluated using Begg’s funnel plot. A two-tailed P value <0.05 was considered statistically significant. All of these statistical analyses were performed by Review Manager Software, version 5.3 (Cochrane, London, UK) and Stata, version 12.0 (Statacorp, College Station, TX).

## 3 Results

### 3.1 Study characteristics

**Figure 1:**
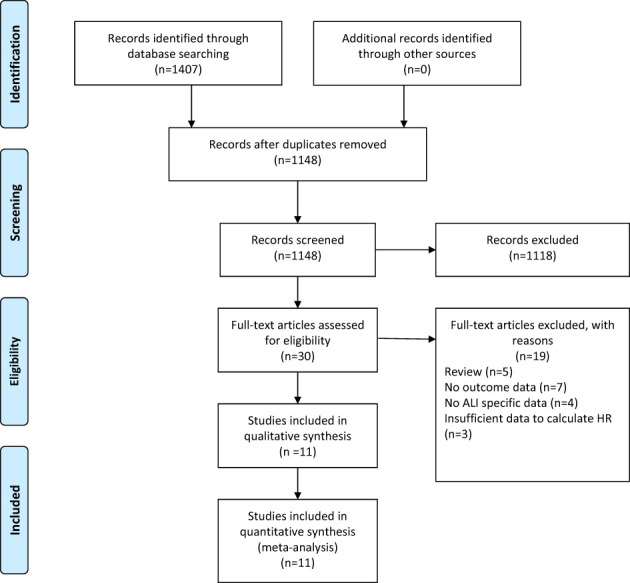
The PRISMA flowchart of study selection.

A flow chart of the selection process was shown in **Figure 1**. The search strategy yielded 1407 potential studies. After title, abstract assessment and full text assessment, 11 retrospective cohort studies (22–32) were finally included in the present study.

The baseline information of the included studies was shown in **Tables 1**, **2**. A total of 4417 patients from China, Korea and Japan were included in this study. These studies were published from 2014 to 2022 with a sample size ranging from 132 to 813. Among these studies, two studies reported esophageal cancer, three studies reported gastric cancer, and six studies reported colorectal cancer. In addition, three studies reported overall POCs, ten studies reported OS, and seven studies reported DFS. Moreover, the cut-off value of the ALI varies a lot among these studies. The details of quality assessment of the included studies were shown in the supplementary file (**Table S1**), and the scores of these studies ranged from 6 to 7 after careful assessment with NOS.

**Table 1:** Basic information of included studies.

Reference	Country	Study design	Study interval	Tumor site	Sample size	Age, years (Median or mean)	Sex (Male/Female)	Tumor stage	Survival analysis	NOS score
Chen,2022 (20)	China	Retro; S	2012-2016	Colorectal cancer	309	61	178/131	Mixed	OS	7
Feng,2014 (21)	China	Retro; S	2006-2008	Esophageal Cancer	293	59.5	259/34	Non-metastatic	CSS	7
He,2022 (22)	China	Retro; S	2009-2014	Gastric cancer	358	61	284/74	Non-metastatic	OS	7
Horino,2021 (23)	Japan	Retro; S	2005-2019	Colorectal cancer	813	NR	464/349	Non-metastatic	OS; RFS	6
Kusunoki,2020 (24)	Japan	Retro; S	2005-2011	Colorectal cancer	298	67	171/127	Mixed	OS; DFS	7
Pian,2020 (25)	Korea	Retro; S	2009-2018	Colorectal cancer	132	62	88/44	Metastatic	OS; DFS	6
Shibutani,2019 (26)	Japan	Retro; S	2008-2016	Colorectal cancer	159	65	87/72	Metastatic	OS	6
Tan,2021 (27)	China	Retro; S	2013-2018	Esophageal Cancer	158	69.5	126/32	Non-metastatic	OS	6
Xie,2020 (28)	China	Retro; S	2012-2014	Colorectal cancer	662	NR	408/254	Mixed	OS; PFS	7
Yin,2020 (29)	Japan	Retro; S	1992-2011	Gastric cancer	620	NR	424/196	Non-metastatic	OS; DFS	7
Zhang,2022 (30)	China	Retro; S	2010-2017	Gastric cancer	615	NR	469/146	Non-metastatic	OS; DFS	7

Retro, retrospective; S, single center; NOS, Newcastle Ottawa Scale; NR, not report.

CSS, cancer specific survival; RFS, recurrence-free survival; PFS, progression-free survival; OS, overall survival; DFS, disease-free survival.

**Table 2:** Survival information of included studies.

Reference	Sample size (High / Low)	Selection method	Cut-off value	Survival analysis	Median follow-up (months)	OS HR (95%CI)	CSS/RFS/PFS/DFS HR (95%CI)
Chen,2022 (20)	309(130/179)	ROC	25.71	OS	60	2.62(1.87-3.66)	NR
Feng,2014 (21)	293(173/120)	Literature	18	CSS	36.8	NR	1.44(1.05-1.96)
He,2022 (22)	358(242/116)	ROC	40.5	OS	101	1.34(0.75-2.44)	NR
Horino,2021 (23)	813(532/281)	classification and regression tree	Male:43.1; Female:13.2	OS; RFS	NR	2.30(1.52-3.50)	1.73(1.22-2.44)
Kusunoki,2020 (24)	298(224/74)	lowest quartile value	20.53	OS; DFS	36.8	3.21(1.97-5.19)	2.13(1.23-3.63)
Pian,2020 (25)	132(32/100)	X-tile	70.4	OS; DFS	NR	2.98(1.32-6.71)	1.46(0.81-2.60)
Shibutani,2019 (26)	159(92/67)	ROC	28.9	OS	21.6	2.77(1.77-4.34)	NR
Tan,2021 (27)	158(57/101)	ROC	31.24	OS	NR	1.86(1.04-3.33)	NR
Xie,2020 (28)	662(423/239)	X-tile	Male:31.6; Female:24.4	OS; PFS	63	1.45(1.11-1.90)	1.37(1.06-1.78)
Yin,2020 (29)	620(449/171)	ROC	30	OS; DFS	NR	1.59(1.15-2.19)	1.26(0.51-3.11)
Zhang,2022 (30)	615(362/253)	ROC	39.77	OS; DFS	NR	1.33(1.01-1.76)	1.36(1.04-1.77)

CSS, cancer specific survival; RFS, recurrence-free survival; PFS, progression-free survival; OS, overall survival; DFS, disease-free survival; NR, not report; ROC, Receiver operating characteristic curve.

### 3.2 Overall survival

**Figure 2:**
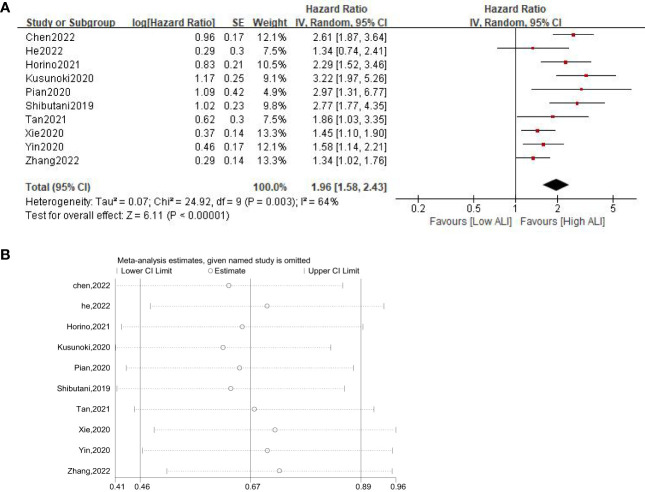
Forest plot **(A)** and sensitivity analysis **(B)** assessing the relationship between ALI and OS.

Ten studies involving 4124 patients (2543 in the high ALI group and 1581 in the low ALI group) reported the OS outcome. The pooled HR was 1.96 (95%CI: 1.58-2.43; P<0.001), which indicated that a low ALI was associated with decreased OS in patients with GI cancer (**Figure 2A**, **Table 3**). Given the presence of moderate heterogeneity (I^2^ = 64%; P=0.003), subgroup analyses based on country, sample size, tumor site, tumor stage, selection method and NOS score were performed. As shown in **Table 3** and **Figure S1**, the pooled results of all subgroup analyses revealed that patients in the high ALI group had a significantly better OS when compared with these in the low ALI group. Additionally, sensitivity analysis by deleting one study at a time showed that the pooled outcome did not substantially change (**Figure 2B**).

**Table 3:** Subgroup analysis for OS of ALI-high patients vs. ALI-low patients.

		Studies, n	Patients, n	HR (95%CI)	P value	I^2^ (%)
	Total	10	4124	1.96 (1.58-2.43)	<0.0001	64
Country	China	5	2102	1.66 (1.26-2.19)	0.0004	64
	Japan	4	1890	2.31 (1.68-3.19)	<0.0001	58
	Korea	1	132	2.97 (1.31-6.77)	<0.0001	–
Sample size	>200	7	3675	1.84 (1.43-2.36)	<0.0001	70
	≤200	3	449	2.48 (1.78-3.44)	<0.0001	0
Selection method	ROC	6	2219	1.84 (1.39-2.42)	<0.0001	65
	Others	4	1905	2.24 (1.47-3.39)	0.0002	70
Tumor site	Esophageal	1	158	1.86 (1.03-3.35)	0.04	–
	Gastric	3	1593	1.42 (1.16-1.73)	0.0006	0
	Colorectal	6	2373	2.36 (1.78-3.14)	<0.0001	64
Tumor stage	Mixed	3	1269	2.24 (1.37-3.66)	0.001	83
	Non-metastatic	5	2564	1.60 (1.31-1.97)	<0.0001	23
	Metastatic	2	291	2.40 (1.86-3.11)	<0.0001	0
NOS score	6	4	1264	2.40 (1.86-3.11)	0.002	0
	7	6	2860	1.78 (1.35-2.35)	<0.0001	72

NOS, Newcastle Ottawa Scale; HR, Hazard ratio; CI, Confidence interval.

### 3.3 Disease-free survival

**Figure 3:**
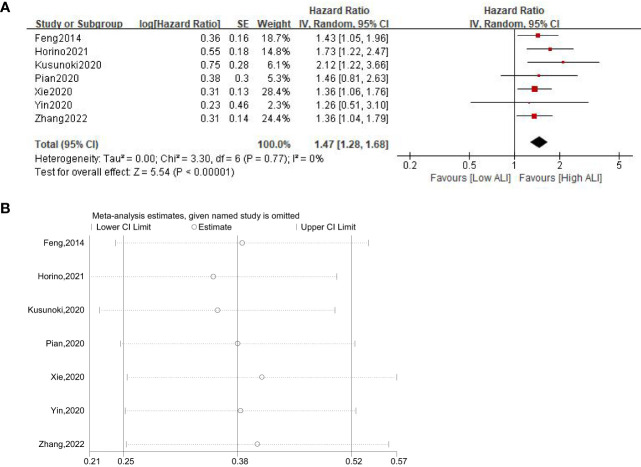
Forest plot **(A)** and sensitivity analysis **(B)** assessing the relationship between ALI and DFS.

A total of seven studies involving 3433 patients (2195 in the high ALI group and 1238 in the low ALI group) reported on DFS. The pooled HR was 1.47 (95%CI: 1.28-1.68; P<0.001; I^2^ = 0%), which suggested that patients in the low ALI group had a worse DFS compared with patients in the high ALI group (**Figure 3A**). Sensitivity analysis showed that the combined effect was not significantly changed (**Figure 3B**).

### 3.4 Postoperative complications

**Figure 4:**
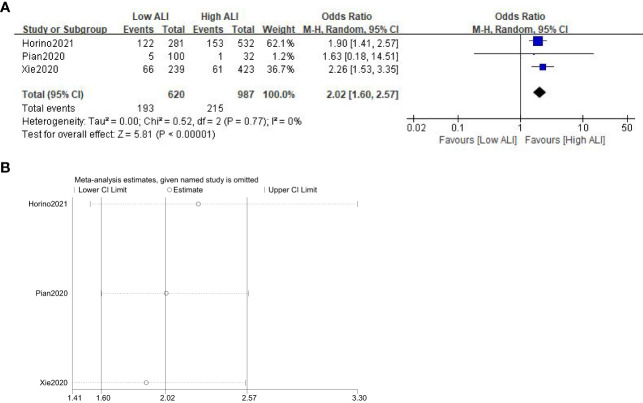
Forest plot **(A)** and sensitivity analysis **(B)** assessing the relationship between ALI and POCs.

Three studies involving 1607 patients (987 in the high ALI group and 620 in the low ALI group) reported this outcome. The pooled OR was 2.02 (95%CI: 1.60-2.57; P<0.001; I^2^ = 0%), which suggested that patients with a low ALI had a higher risk of POCs than those with a high ALI (**Figure 4A**). Similarly, sensitivity analysis did not show significant change for the pooled outcome (**Figure 4B**).

### 3.5 Publication bias

**Figure 5:**
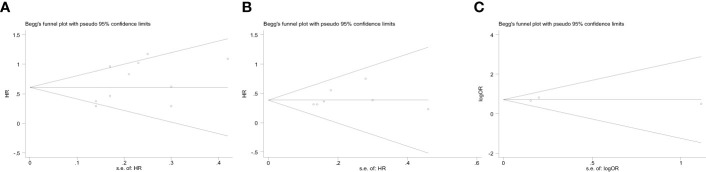
Begg’s funnel plot assessing publication bias between ALI and therapeutic outcomes, including **(A)** OS, **(B)** DFS, **(C)** POCs. The Begg’s P values were 0.239, 0.230, and 1.000, respectively.

The Begg’s funnel plot was performed to assess the potential publication bias. As shown in **Figure 5**, the funnel plots of OS, DFS and POCs were virtually symmetric, and the P values of Begg’s test were 0.239, 0.230, and 1.000, respectively, indicating that these pooled outcomes had a low risk of publication bias.

## 4 Discussion

In 2013, Jafri et al. (13) first established the ALI based on commonly used clinical parameters as a systemic inflammation-related prognostic score tool for metastatic non-small cell lung cancer. Since then, the ALI has been widely used as a readily available and reliable biomarker to evaluate the prognosis of coronary artery disease (33), lung cancer (34) and pancreatic cancer (35). As shown in **Table 1**, most of included studies were published after 2019, indicating that the ALI is still a very new index in the field of GI cancer.

In this study, we included eleven studies with 4417 patients with GI cancer and found that a low ALI was significantly associated with decreased OS (HR=1.96; 95%CI: 1.58-2.43; P<0.001) and DFS (HR=1.47; 95%CI: 1.28-1.68; P<0.001). Meanwhile, we have further identified that the ALI could also act as a predictor for POCs (OR=2.02; 95%CI:1.60-2.57; P<0.001) in patients with GI cancer. Therefore, the ALI may have a good discriminatory value and remains an effective inflammatory/nutrition factor for predicting therapeutic outcomes in GI cancer.

Systemic inflammatory reflection (SIR) is recognized as the 7th hallmark of cancer which is closely associated with the occurrence and progression of malignancies (36). Consistent with this evidence, the SIR as a potential prognostic marker is also demonstrated for various malignancies, and the NLR is one of the well-established SIR markers (37, 38). Increased neutrophils in the tumor microenvironment have been reported to prompt the tumor growth and metastasis by releasing chemokines and cytokines (39). Besides, neutrophils can also inhibit the activation of T lymphocytes, thereby inhibiting the anti-tumor immunity of the host (39, 40). While lymphocytes, especially CD4+ T lymphocytes and CD8+ T lymphocytes, as the most important immune cells, play an anti-tumor role by inducing the lysis and apoptosis of tumor cells (41). Lymphopenia has been demonstrated to be associated with poor prognosis in cancer patients (42, 43). On the other hand, nutritional status is also reported as an important predictive factor of therapeutic outcomes in several types of malignancies (44, 45). Studies have proven that malnutrition leads to an inadequate anti-tumor immune response and reduces wound healing ability, thereby reducing treatment efficacy and leading to severe POCs (46, 47). As an objective and common measurement reflecting patients’ nutritional status, baseline BMI and ALB have been indicated to be positively associated with the short-term and long-term outcomes of cancer patients and have been used to triage patients in clinic care (48). Reasonably, the ALI, combined with these factors, is a useful comprehensive indicator of nutritional and inflammatory status, may enable better understanding of the functional state of patients and predict the therapeutic results of patients with GI cancer.

In our pooled analysis involving 4124 participants, we found that the ALI is an independent factor influencing the OS of patients with GI cancer. Even though significant heterogeneity existed, our subgroup analyses based on country, sample size, tumor site, selection method, tumor stage and NOS score showed our results were reliable and robust. Meanwhile, the sensitivity analysis showed that there was no change of the evident correlation between low ALI and poor OS. In addition, we have further investigated the relationship between the ALI and DFS. The combined result including 3433 patients showed that a low ALI was significantly associated with decreased DFS in GI cancer patients without obvious heterogeneity. Likewise, the sensitivity analysis supported the consistence and reliability of the result. We have also explored the correlation between ALI and POCs. The integrated result demonstrated that low ALI could act as a predictor of the incidence of POCs without heterogeneity. Furthermore, sensitivity analysis suggested that the result of meta-analysis for POCs was reliable. Based on these results, the ALI may be regarded as an effective clinical indicator predicting the short-term and long-term results of GI cancer patients.

There are some limitations in our study. First, all of the involved studies were retrospective studies performed at a single center, which may increase the risk of bias, and more prospective studies are required to further investigate this issue. Second, all included studies are Asian cohorts and studies from western countries are lacking, which may lead to potential publication bias and limit the generalization of the results. Third, due to the limited number of included studies, the value of the ALI in POCs, especially in specific POCs, needs to be further clarified. Finally, the cut-off value of the ALI among studies varied a lot, which might affect the validity and clinical utility of these findings; and further studies that use ALI as a continuous variable are warranted to verify this issue.

## 5 Conclusions

The findings of the meta-analysis suggested that the ALI prior to initial treatment is of great significance in predicting POCs and long-term survival results in patients with GI cancer. However, high-quality prospective studies with large sample size are still required to further validate the value of ALI in GI cancer.

## Data availability statement

The original contributions presented in the study are included in the article/**Supplementary Material**. Further inquiries can be directed to the corresponding author.

## Author contributions

H-YP wrote the manuscript. H-YP, X-FC and M-HY performed the data search and data analysis. H-YP, M-HY and L-HC prepared figures. All authors reviewed the manuscript. HS approved the final manuscript. All authors contributed to the article and approved the submitted version.
