# Supplementary material for: Clinical significance of the advanced lung cancer inflammation index in gastrointestinal cancer patients: a systematic review and meta-analysis
Source: Front Oncol. 2023 Jun 19;13:1021672. doi: 10.3389/fonc.2023.1021672 (PMC10316012; doi:10.3389/fonc.2023.1021672)
Supplement: Supplementary file 1 [file DataSheet_1.docx]

Table S1. Quality assessment of included studies using Newcastle-Ottawa Scale.

| **Cohort study** | **Representatives of the exposed cohort** | **Selection of the non-exposed cohort** | **Ascertainment of exposure** | **Was outcome of interest present at start of study** | **Comparability of cohorts on the basis of the design or analysis** | **Assessment of outcome** | **Was follow-up long enough for outcomes to occur** | **Adequate follow up** | **Total** |
| --- | --- | --- | --- | --- | --- | --- | --- | --- | --- |
| Chen,2022[20] | 1 | 1 | 1 | 0 | 0 | 1 | 1 | 1 | 7 |
| Feng,2014[21] | 1 | 1 | 1 | 0 | 0 | 1 | 1 | 1 | 7 |
| He,2022[22] | 1 | 1 | 1 | 0 | 0 | 1 | 1 | 1 | 7 |
| Horino,2021[23] | 1 | 1 | 1 | 0 | 0 | 1 | 0 | 1 | 6 |
| Kusunoki,2020[24] | 1 | 1 | 1 | 0 | 0 | 1 | 1 | 1 | 7 |
| Pian,2020[25] | 1 | 1 | 1 | 0 | 0 | 1 | 0 | 1 | 6 |
| Shibutani,2019[26] | 1 | 1 | 1 | 0 | 0 | 1 | 0 | 1 | 6 |
| Tan,2021[27] | 1 | 1 | 1 | 0 | 0 | 1 | 0 | 1 | 6 |
| Xie,2020[28] | 1 | 1 | 1 | 0 | 0 | 1 | 1 | 1 | 7 |
| Yin,2020[29] | 1 | 1 | 1 | 0 | 0 | 1 | 1 | 1 | 7 |
| Zhang,2022[30] | 1 | 1 | 1 | 0 | 0 | 1 | 1 | 1 | 7 |


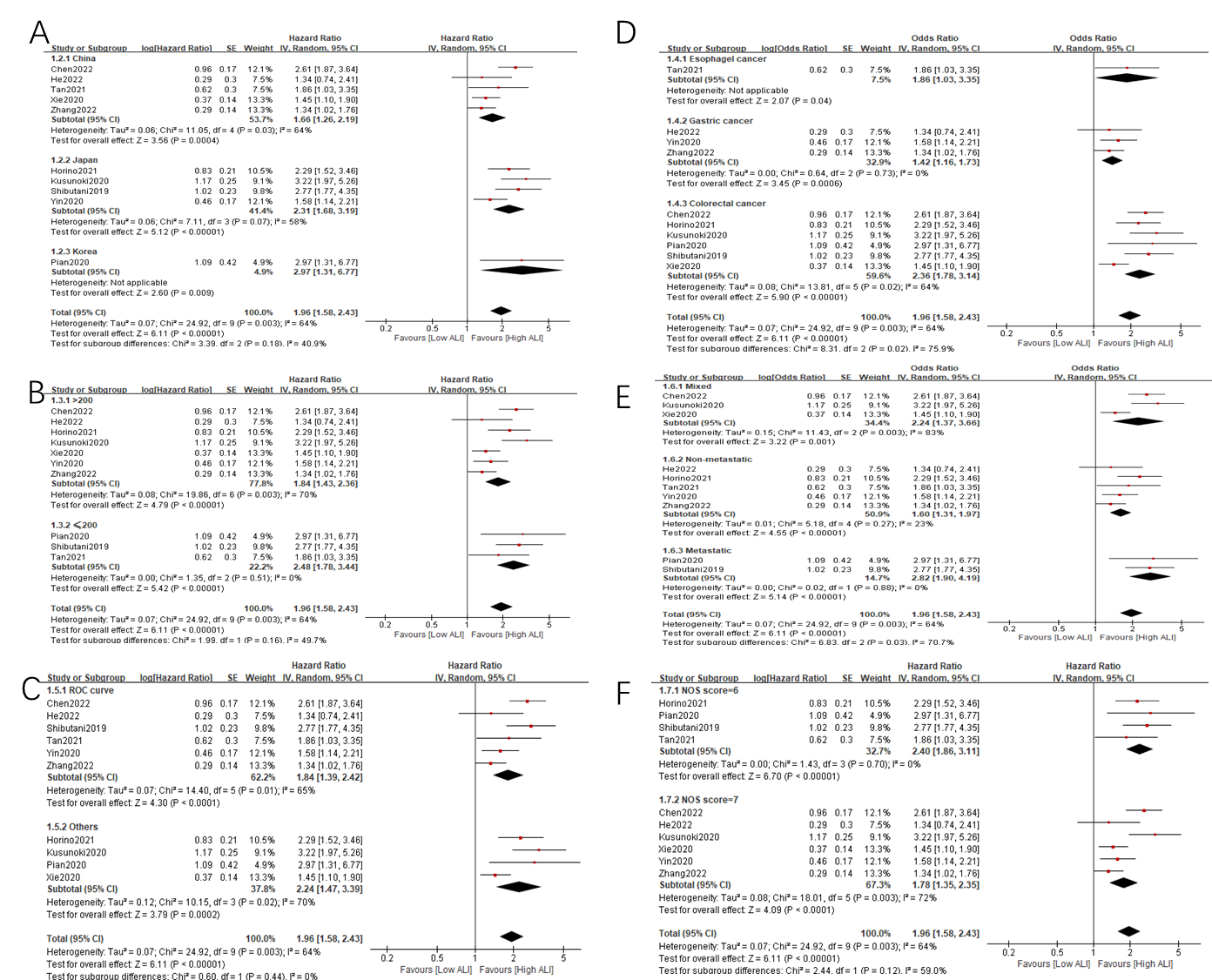


Figure S1. Forest plot of subgroup analyses assessing the relationship between ALI and OS. A: Country (China vs. Korea vs. Japan); B: Sample size (>200 vs. ≤200); C: Selection method (ROC curve vs others); D: Tumor stage (non-metastatic vs. metastatic vs. mixed); E: Tumor site (Esophageal vs. Gastric vs. Colorectal); F: NOS score (Score 6 vs. Score 7).
